# Lethal Caspase-1/4-Dependent Injury Occurs in the First Minutes of Coronary Reperfusion and Requires Calpain Activity

**DOI:** 10.3390/ijms24043801

**Published:** 2023-02-14

**Authors:** Xi-Ming Yang, Michael V. Cohen, Sarah Sayner, Jonathon P. Audia, James M. Downey

**Affiliations:** 1 Department of Physiology and Cell Biology, College of Medicine, University of South Alabama, Mobile, AL 36688, USA; 2 Department of Medicine, College of Medicine, University of South Alabama, Mobile, AL 36688, USA; 3 Department of Microbiology, College of Medicine, University of South Alabama, Mobile, AL 36688, USA

**Keywords:** AC10, cardioprotection, calpain, caspase-1, emricasan, VRT-043198

## Abstract

To study the relationship between caspase-1/4 and reperfusion injury, we measured infarct size (IS) in isolated mouse hearts undergoing 50 min global ischemia/2 h reperfusion. Starting VRT-043198 (VRT) at reperfusion halved IS. The pan-caspase inhibitor emricasan duplicated VRT’s protection. IS in caspase-1/4-knockout hearts was similarly reduced, supporting the hypothesis that caspase-1/4 was VRT’s only protective target. NLRC4 inflammasomes activate caspase-1. NLRC4 knockout hearts were not protected, eliminating NLRC4 as caspase-1/4’s activator. The amount of protection that could be achieved by only suppressing caspase-1/4 activity was limited. In wild-type (WT) hearts, ischemic preconditioning (IPC) was as protective as caspase-1/4 inhibitors. Combining IPC and emricasan in these hearts or preconditioning caspase-1/4-knockout hearts produced an additive IS reduction, indicating that more protection could be achieved by combining treatments. We determined when caspase-1/4 exerted its lethal injury. Starting VRT after 10 min of reperfusion in WT hearts was no longer protective, revealing that caspase-1/4 inflicted its injury within the first 10 min of reperfusion. Ca^++^ influx at reperfusion might activate caspase-1/4. We tested whether Ca^++^-dependent soluble adenylyl cyclase (AC10) could be responsible. However, IS in AC10^−/−^ hearts was not different from that in WT control hearts. Ca^++^-activated calpain has been implicated in reperfusion injury. Calpain could be releasing actin-bound procaspase-1 in cardiomyocytes, which would explain why caspase-1/4-related injury is confined to early reperfusion. The calpain inhibitor calpeptin duplicated emricasan’s protection. Unlike IPC, adding calpain to emricasan offered no additional protection, suggesting that caspase-1/4 and calpain may share the same protective target.

## 1. Introduction

Sterile inflammation arising from the innate immune system has recently been recognized as a contributor to necrosis in acute myocardial infarction. Ischemic myocardium releases chemicals, such as ATP, potassium, and oxidized mitochondrial DNA. These damage-associated molecular patterns (DAMPs) can be sensed by molecular receptors, such as NLRP3 (NOD-like receptor pyrin domain-containing protein 3). Activated NLRP3 protein combines with two other proteins, apoptosis-associated speck-like protein containing CARD (ASC) and procaspase-1, to form the NLRP3 inflammasome. In the process, procaspase-1 is proteolytically converted to its active moiety, caspase-1. Active caspase-1 proteolytically cleaves gasdermin D, and its N-terminal fragment forms large pores in the cardiomyocyte’s sarcolemma. Pore formation permits the secretion of proinflammatory interleukins that have been activated by caspase-1. However, this loss of membrane integrity, termed pyroptosis, sacrifices the cell. This process is designed to combat invasion by pathogens but is also inappropriately triggered by DAMPs released during ischemia/reperfusion (I/R) in the sterile heart. We [1] and others [2,3] have observed that suppression of inflammasome formation or their activated caspase-1 protects animal myocardial I/R models against infarction.

We previously studied VX-765, a prodrug, whose active product, VRT-043198 (VRT), is a highly selective inhibitor of human caspase-1 and caspase-4 [4]. VX-765, which has been approved for human investigation (clinicaltrials.gov NCT01048255 and NCT00205465, [5]), is rapidly converted to VRT by esterases in the blood [6]. Administering VX-765 to open-chest rats at reperfusion [1] is just as protective as administering VX-765 prior to ischemia [7], indicating that caspase-1 (and/or 4) causes all of its injury during reperfusion. VX-765 is ineffective in isolated buffer-perfused rat hearts because Krebs buffer is unable to convert VX-765 to VRT. However, VRT at reperfusion in isolated rat hearts was just as protective as VX-765 in in situ hearts [1]. These experiments indicate that all of the components responsible for infarction that VRT targets reside wholly within the rat heart and therefore exclude any role for circulating inflammatory cells. In addition, the size of infarcts and their salvage caused by the caspase-1/4 inhibitor in isolated rat hearts after only 2 h of reperfusion was very similar to that found in in situ rat hearts experiencing either 2 or 72 h of reperfusion, which argued against any appreciable late cell killing [1]. At least in the rat, the ultimate infarct size was reached after only 2 h of reperfusion, making the blood-free isolated heart a clinically relevant model for studying the pharmacology of caspase-1/4 blockers. 

Attempts to limit infarct size by ischemic postconditioning, a process that is very effective in animal models, in patients undergoing interventions for acute coronary thrombosis have been disappointing [8]. This failure may be the result of aspirin loading [9]. Protection against infarction by VX-765 can be added to protection from ischemic postconditioning in rat hearts, implying that these two interventions protect by two simultaneous and independent mechanisms [1,7]. Because of the differing mechanisms of protection, caspase-1/4 inhibition may not be affected by aspirin and, if so, would be a candidate for clinical testing. 

We used isolated murine hearts for our study. We initially tested whether caspase-1 inhibition in the isolated mouse heart is as protective against myocardial infarction as it is in the rat heart. We observed that the protective effect of caspase-1 inhibition is, indeed, not unique to rat hearts.

The time course of caspase-1 cell killing in the reperfused heart is controversial. In one published study, active caspase-1 measured by Western blot analysis appreciably increased in mouse hearts only after 20 min of reperfusion [3], while in another study, an NLRP3 inhibitor was just as protective when started after 1 h of reperfusion as when started at the onset of reperfusion [10]. Our previous rat studies revealed that caspase-1 inhibition prevented injury during reperfusion and that most of the LDH released from reperfused isolated rat hearts occurred during the first 5 min of reperfusion [1]. By delaying the start of the VRT infusion, we hoped to identify a time point at which VRT’s protection would be lost. If such a point could be found, then we could conclude that caspase-1-dependent lethal reperfusion injury had been completed by that time. 

We also wanted to critically test whether caspase-1/4 was VRT’s protective target. First, we tested whether a chemically different caspase-1/4 inhibitor, emricasan (also known as IDN 6556), could duplicate VX-765′s or VRT’s protection against infarction. Emricasan is a pan-caspase antagonist [11] and should therefore block caspase-1 and caspase-4. Therefore, we tested whether exposing isolated mouse hearts to emricasan would duplicate protection from VRT. Additionally, we wanted to test whether protection from VRT could be duplicated in the hearts of genetically modified mice in which caspase-1 and caspase-4 were absent.

We wanted to determine why caspase-1/4 is only activated at reperfusion and whether the rise in cytosolic Ca^++^ which occurs at the onset of reperfusion might be responsible. Ca^++^ has long been implicated in I/R injury in the heart, although its mechanism of lethality has never been identified. One candidate for a calcium-dependent mediator of cell killing is soluble adenylyl cyclase (AC10). AC10 is activated by Ca^++^ and bicarbonate ions [12], which is in contrast to membrane-bound adenylyl cyclases, which are G-protein-coupled. Myocardial reperfusion is accompanied by a dramatic rise in tissue pH and elevated Ca^++^. Indeed, one recent study reported that AC10 contributed to apoptosis in cardiomyocytes experiencing simulated I/R [13]. In contrast, another study reported that overexpression of AC10 protected isolated cells [14]. To resolve this issue, we proposed measuring infarct size in AC10-deficient hearts. 

Ca^++^-activated calpain during early reperfusion is another possible activator of caspase-1/4 activity at reperfusion. Ca^++^-activated calpain in macrophages releases actin-bound procaspase-1, which is required to complete the assembly of the NLRP3 inflammasome [15]. Calpain in the heart is activated by Ca^++^ influx during the first few minutes of reperfusion. Inhibition of calpain has been reported to reduce infarct size in a number of models [16,17,18], but the exact mechanism has never been confirmed. If caspase-1 is actin-bound in the cardiac muscle, then calpain inhibition could block its release at reperfusion. Therefore, we tested whether a calpain inhibitor could mimic the protection of caspase-1 inhibitors in our mouse heart model.

## 2. Results

There were no differences in baseline coronary flow among the groups. During early reperfusion, flow decreased by 25–50% in all groups. During the reperfusion period, there was either no decrease in flow or a gradual small further decrease.

**Figure 1:**
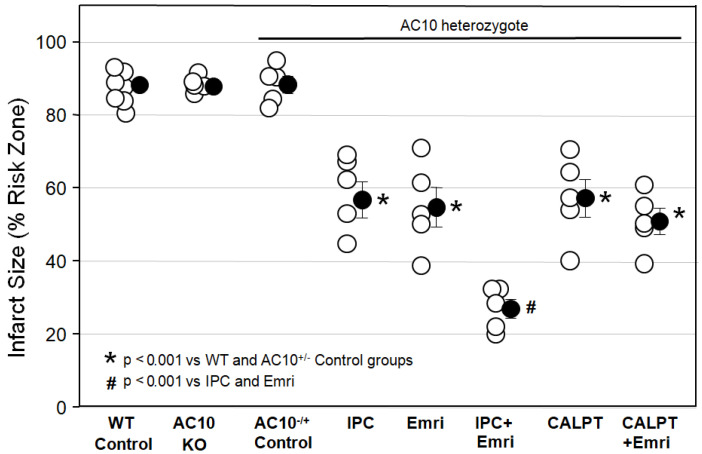
Infarct size as a percentage of risk zone in isolated male mouse hearts undergoing 50 min global ischemia/2 h reperfusion. Open circles represent individual hearts, and solid circles represent means ± SEM. Abbreviations: AC10, soluble adenyl cyclase; CALPT, calpeptin; Emri, emricasan; IPC, ischemic preconditioning; KO, knockout; WT, wild type.

Figure 1 shows that infarct sizes among Krebs-perfused AC10^−/−^ KO hearts (AC10 KO), WT hearts (WT Control), and AC10^−/+^ heterozygous (AC10^−/+^ Control) mouse hearts with 50 min of global ischemia and 2 h of reperfusion were not different, indicating that AC10 does not play a protective or detrimental role in the heart during I/R. Because their infarct size was not different from that in WT mice, many of the surplus AC10^−/+^ mice that were generated in the breeding program that was used to generate AC10^−/−^ knockouts were used for several of the cohorts in protocol I. The goal was to greatly reduce the number of mice sacrificed in this study.

Figure 1 shows that ischemic preconditioning (IPC) caused a marked reduction in infarct size, as would be expected. Emricasan (Emri) for the first 20 min of reperfusion was equally as protective as IPC, indicating that a caspase was contributing to the infarction. Emricasan is an irreversible inhibitor of all caspases, including caspase-1 and caspase-4. In addition, since emricasan was present only during reperfusion, it must have prevented reperfusion injury. When we combined emricasan with IPC, the infarct size was significantly smaller than that with either IPC or emricasan alone. The additive protection suggests that two differing mechanisms of cell killing were simultaneously contributing to infarction. 

We next tested the calpain inhibitor calpeptin (CALPT). Calpeptin was present in the buffer throughout the study. Calpeptin was just as protective as emricasan, indicating that calpain activity also adds to the infarction process. Unlike the increased protection seen with the combination of IPC and emricasan, combining calpeptin and emricasan showed no additive effect. This suggests that calpain activity plays a critical role in the same lethal mechanism that caspase-1/4 does. 

**Figure 2:**
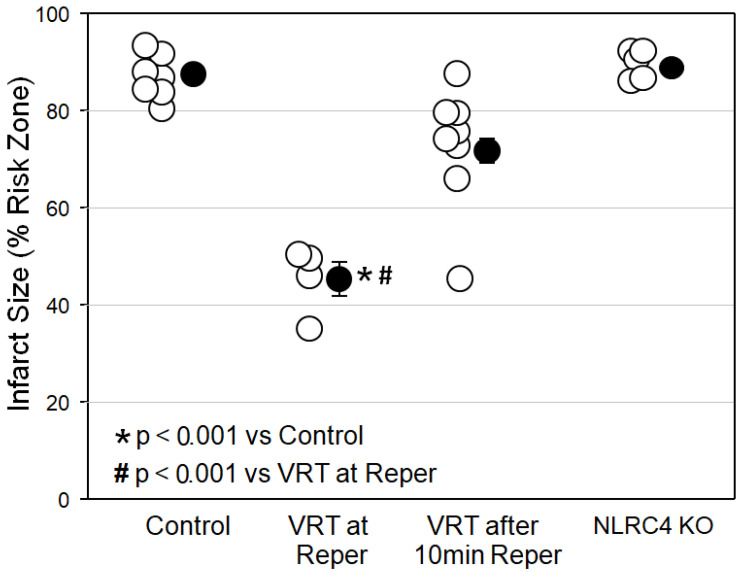
Infarct size as a percentage of the risk zone in isolated male wild-type and NLRC4-KO mouse hearts undergoing 50 min global ischemia/2 h reperfusion. Open circles represent individual hearts, and solid circles represent means ± SEM. Abbreviations: KO, knockout; Reper, reperfusion; VRT, VRT-043198.

Figure 2 reveals that VRT at reperfusion in isolated mouse hearts was just as protective as emricasan at reperfusion had been in Figure 1. However, when we delayed the VRT treatment until the heart had been reperfused for just 10 min, all of VRT’s protection was lost, indicating that caspase-1/4 causes most, if not all, of its injury in the first 10 min of reperfusion. Finally, we tested hearts from mice in which the NLRC4 inflammasome had been knocked out. The NLRC4 inflammasome, when activated by lipopolysaccharides, can also activate caspase-1. These hearts showed no protection, however, thus eliminating the NLRC4 inflammasome as a possible source of caspase-1/4 activity.

**Figure 3:**
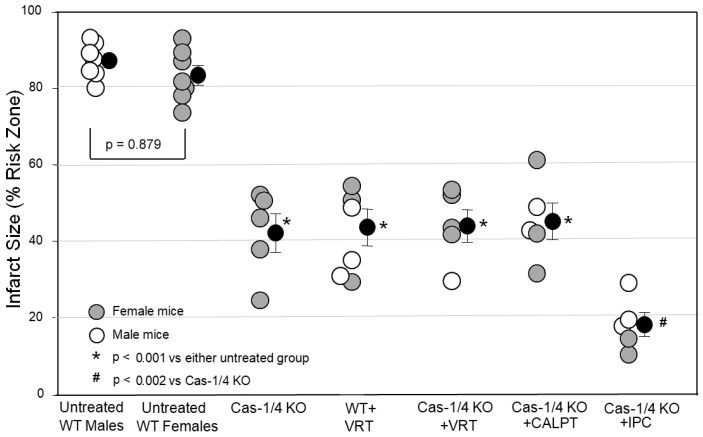
Infarct size as a percentage of the risk zone in isolated mouse hearts undergoing 50 min global ischemia/2 h reperfusion. Open circles represent individual hearts, and solid circles represent means ± SEM. Abbreviations: CALPT, calpeptin; Cas-1/4, Caspase-1/4; IPC, ischemic preconditioning; KO, knockout; VRT, VRT-043198; WT, wild type.

A major aim of this study was to critically test whether caspase-1/4 was the protective target of VX-765 and VRT. Using pharmacology, there is always the possibility that VX-765 and VRT were actually protecting by targeting something other than caspase-1. We partially approached this problem by testing whether another caspase-1 inhibitor, emricasan, could duplicate VX-765’s and VRT’s protection, which it did. The ultimate test, however, would be to see if VRT could protect a heart in which caspase-1 had been genetically disabled. In Figure 3, we tested hearts from caspase-1/4-KO mice. Infarct sizes in the caspase-1/4-deficient hearts were as small as those in WT hearts treated with the caspase-1 blocker VRT. This would be expected if caspase-1/4 were the target of the inhibitors, but the protection could also have resulted from some nonspecific change in these genetically altered mice unrelated to caspase-1/4. Therefore, we tested VRT in the caspase-1/4-deficient hearts. While the infarcts in wild-type hearts treated with VRT were just as small as those in caspase-1-KO hearts, treating caspase-1-KO hearts with VRT caused no additional protection, confirming that caspase-1/4 was indeed VRT’s only protective target. 

We saw in Figure 1 that the calpain inhibitor calpeptin duplicated the protection of caspase blockers but, when combined with a caspase blocker, offered no additional protection, suggesting that calpain and caspase-1/4 blockers were providing protection using the same mechanism. Treating caspase-1-KO hearts with calpeptin offered no additional protection either (Figure 3). However, additive protection was seen when the caspase-1-KO hearts were ischemically preconditioned. We interpret this to mean that IPC targets a mechanism of cell killing that is operating independently of and in parallel with that involving caspase-1/4. On the other hand, both calpain and caspase-1 activity appear to be critical components of the same mechanism of cell killing. 

## 3. Discussion

Initially, we wanted to evaluate the isolated, buffer-perfused mouse heart as a model for evaluating caspase-1/4 inhibitors as cardioprotectants. In 2009, Lacerda et al. [19] reported experiments using a very similar isolated mouse heart preparation. All hearts throughout our study were examined using the same color threshold settings. We found that mouse hearts exposed to 50 min of global ischemia and 2 h of reperfusion yielded very reproducible infarct sizes. Rapid cannulation of the tiny hearts is technically challenging. Therefore, our protocol required that we studied only hearts that were reperfused with buffer within 4 min of being excised from the anesthetized mouse to avoid preconditioning. Preparations in which cannulation time exceeded 4 min were not studied. This model has several advantages. Foremost, it allows the use of readily available, genetically altered murine hearts. Another advantage is that drugs can be administered in the perfusate at precisely known concentrations and at precise times. The isolated mouse heart has a very slow flow rate of buffer (approximately 1–2 mL/min), which greatly reduces the amount of drug needed for an experiment. Since the created ischemia is global, the entire heart becomes the risk zone, thus eliminating the need to identify a tiny risk zone by staining the nonischemic tissue with dye or microspheres. Furthermore, chemical assays of the ischemic tissue can be achieved accurately by simply homogenizing the entire heart. Finally, we found that our caspase inhibitors were equally as protective against infarction in mouse hearts (Figure 1 and Figure 2) as they were in rat hearts [1,7], thus validating the mouse heart for mechanistic studies of cardioprotective caspase inhibitors. 

Our next objective was to determine the timing of the VRT-sensitive injury. In the in situ rat heart, VRT’s prodrug VX-765 at reperfusion [1] was no more protective than when administered prior to ischemia [7], indicating that VRT’s target is causing all of its lethal injury during reperfusion. Since starting VRT at reperfusion greatly reduced infarct size, it must have done so by eliminating reperfusion injury. In the present study (Figure 2) and in our previous rat experiments [1], VRT was present in the buffer throughout reperfusion. Therefore, it was not possible to determine when VRT exerted its protective effect during the 2 h reperfusion period. However, when we started VRT after 10 min of reperfusion in the present study, no significant reduction in infarct size occurred (Figure 2). Therefore, all of the VRT-dependent infarction must have occurred in the first 10 min of reperfusion. The isolated rat heart releases a large quantity of LDH in the first minutes of reperfusion, and VRT greatly attenuates that release, implying that injury is produced during those initial minutes of reperfusion [1]. Those isolated rat heart data are consistent with the present observations in murine hearts. 

VRT targets caspase-1 and caspase-4, which are thought to be activated by inflammasomes and which are thought to do their cell killing by proteolytic degradation of gasdermin D, resulting in production of the N-terminal fragment, which then destroys the cell by making large pores in its outer membrane [20]. This process is termed pyroptosis and was confirmed when gasdermin D-deficient hearts were shown to be protected against infarction [21].

Not all studies agree with our findings. Toldo et al. [10] also evaluated the critical timing of myocardial necrosis and drug-mediated salvage in open-chest mouse hearts with regional ischemia. They saw a time-dependent increase in infarct size measured at 1, 3, and 24 h after reperfusion. An NLRP3 inflammasome inhibitor, 16673-34-0, was very protective when injected at reperfusion and infarct size was measured 24 h later [22]. When they delayed its injection until 1 h after reperfusion, it was still protective. However, when they injected it 3 h after reperfusion, protection was lost. They concluded that the NLRP3-dependent injury occurred somewhere between 1 and 3 h after the onset of reperfusion, which does not agree with the present findings using a caspase-1/4 inhibitor. 

Kawaguchi et al. [23] reported that NLRP3 protein is constitutively expressed in cardiac fibroblasts but not in myocytes. Bone marrow transplantation experiments in genetically manipulated mice revealed that inflammasome activation in both bone marrow cells and myocardial resident cells contributes to myocardial infarction during I/R. The authors concluded that inflammasomes in the heart’s fibroblasts acted to attract leukocytes, and it was the leukocytes that actually killed the heart tissue. That scenario is obviously incompatible with the results in the present model in which blood components were absent. The infarcts we saw in both our in situ and isolated rat hearts [1,7] were very similar, suggesting that circulating blood components, such as leukocytes, do not significantly contribute to caspase-1/4’s lethality.

The role of NLRP3 in myocardial infarction is controversial. Mastrocola et al. [3] observed infarct size reduction with an NLRP3 inhibitor in isolated rat hearts. Genetic deletion of NLRP3 failed to reduce infarct size in a closed-chest in situ mouse heart model (30 min ischemia/3 h reperfusion), which was attributed to its low expression in heart tissue [24]. Finally, Sandanger et al. [25] actually saw an increased infarct size in their NLRP3-KO mice. We did not examine NLRP3’s role in the present study.

In a recent study [1], we subjected in situ rat hearts to 60 min of regional ischemia and 2 h of reperfusion. Some were treated at reperfusion with VX-765 and the platelet inhibitor cangrelor. When given separately, these two agents were equally protective, and when combined, their protection was additive. We repeated the experiments in control open-chest rats and rats treated with the combination of VX-765 and cangrelor and extended the reperfusion period to 3 days. Infarct sizes in both the control and treated rats were virtually identical to those seen in rats with only 2 h of reperfusion. Thus, we could not find any evidence of delayed infarction between 2 and 72 h of reperfusion in the in situ rat hearts. However, we have not similarly examined in situ mouse hearts. 

Many studies of the biochemistry of reperfusion injury have been obtained from hearts that had been subjected to ischemia plus several hours of reperfusion. In contrast, our data reveal that the lethal effect of caspase-1/4 activation must have occurred in the first 10 min of reperfusion, and we suggest that biochemical measurements should be made in these early minutes as well. Infarcted tissue becomes tetrazolium negative after several hours of reperfusion because the cardiomyocyte’s dehydrogenase enzymes wash out through defects in the cell membrane, and the same should be true for all soluble proteins. Therefore, analysis of infarcted tissue after several hours of reperfusion is unlikely to yield an accurate snapshot of the critical early minutes of reperfusion. Furthermore, when studying the inflammatory component in in situ hearts, the investigator should be aware that leukocytes soon begin to accumulate; these cells contain many inflammation-related proteins but obviously had nothing to do with the very early cardiomyocyte killing caused by caspase-1/4. 

Caspase-mediated injury is not the only injury that occurs in early reperfusion. Adenosine agonists given at reperfusion protect through the conditioning pathway. The adenosine A_1_/A_2_ receptor agonist AMP579 started at reperfusion greatly reduced infarct size, but it was no longer protective when started 10 min after reperfusion [26]. Moreover, the anti-infarct effect of cangrelor, a P2Y_12_ receptor inhibitor that blocks platelet aggregation, administered just before reperfusion was lost when administered after 10 min of reperfusion [27]. That same study found strong evidence that cangrelor protects through the conditioning pathway. Since conditioning and a caspase inhibitor can provide additive protection, they presumably target differing mechanisms of cell killing. Thus, it appears that at least two mechanisms of lethality occur in the first few minutes of reperfusion. Importantly, our timing studies reveal that any cardioprotective intervention designed to mitigate reperfusion injury would have to be in place prior to the onset of reperfusion to ensure treatment before the injury has occurred. 

It should be noted that one recent publication by Do Carmo et al. [28] reported that additive protection was not seen when VX-765 and ischemic preconditioning were combined. We saw a robust additive effect of a caspase inhibitor and IPC in both the present murine study (Figure 1) and in our previous isolated rat heart study [1]. A major difference between our model and that of Do Carmo et al. is that they used only 30 min of ischemia, whereas we used a much longer insult. With only 30 min of ischemia, infarct size is already near zero with either single intervention; therefore, further infarct size reduction from a second intervention may be difficult to detect. 

VRT was found to be highly selective against human caspase-1 and caspase-4 [4]. Mice, as used in the present study, do not express a homolog of human caspase-4 but do express caspase-11, which is often referred to as caspase-4 in the literature because it, like human caspase-4, is activated primarily by lipopolysaccharides [29]. We could not find any information about whether VRT can block murine caspase-11. However, caspase-11 is not constitutively expressed in most murine tissues and is only thought to be expressed in response to lipopolysaccharides [29]. Thus, it would seem unlikely that caspase-11 would even be present during the sterile inflammation of acute myocardial I/R. Recently, Shi et al. [21] studied a model of pyroptosis in isolated murine cardiomyocytes exposed to hypoxia/reoxygenation to simulate I/R. Pyroptosis was seen in WT and caspase-1-deficient cardiomyocytes, but it was absent in caspase-11-deficient cells. Thus, Shi et al. concluded that caspase-11 rather than caspase-1 was causing pyroptosis. Whether caspase-11-deficient hearts would also be protected from infarction is unknown. Kawaguchi et al. [23], as in the present study, reported that their strain of caspase-1-deficient hearts was protected against infarction. We do not know whether caspase-4 was also absent in those hearts. Thus, the role of caspase-4 remains unresolved.

One way to test for a nonspecific effect of a drug is to determine whether a second known inhibitor of the same suspected target can duplicate the primary action. We tested emricasan, which greatly differs chemically from VRT. Unlike VRT, emricasan is an irreversible blocker of all caspases, which would include caspase-1/4. Emricasan was as protective as VRT (Figure 1, Figure 2 and Figure 3). This result is interesting because caspases, such as caspase-3, caspase-6, and caspase-7, have been implicated in infarction through apoptosis when they cause cells to die in response to destruction of their DNA [20]. There was no indication that emricasan was any more protective than either VRT at reperfusion in these mouse hearts (Figure 1 and Figure 2) or VX-765 administered as pretreatment in rat hearts [7]. Thus, it would appear that emricasan not only duplicates VRT’s protection, but removing the triggers for apoptosis does not offer any further protection in our acute model. Theoretically, an irreversible inhibitor of caspases briefly infused into the ischemic zone prior to the recanalization of patients receiving primary percutaneous angioplasty might allow local, persistent, and targeted cardioprotective caspase-1/4 inhibition. Emricasan, like VX-765, has been granted IND status for use in clinical trials [30]. 

What causes caspase-dependent cell killing to occur only during early reperfusion? Two important events occur at that time: production of reactive oxygen species and Ca^++^ influx. To test the latter, we performed two experiments. We investigated the possible role of the calcium-dependent adenylyl cyclase AC10. AC10 reportedly contributed to apoptosis in cardiomyocytes experiencing simulated I/R [13], while a second study found that overexpression of AC10 actually protected isolated cells [14]. However, we found that its genetic deletion had no effect on infarct size in our mouse hearts (Figure 1), indicating that AC10 was not involved. 

Next, we tested whether the calcium-dependent protease calpain plays a role. Inhibition with calpeptin was as protective as VRT (Figure 1 and Figure 3), but either combining it with emricasan (Figure 1) or treating caspase-1/4- deficient hearts with it (Figure 3) offered no additional protection, suggesting that calpain targets the same anti-infarct mechanism as caspase-1/4. Although calpain inhibitors have previously been reported to protect against infarction [16,17,18], their protective mechanism has never been documented. In a recent study of bone marrow-derived macrophages, calpain was shown to release procaspase-1, which is normally bound to cytoskeletal actin by flightless-1 protein [15]. Calpain activation by Ca^++^ influx frees bound procaspase-1, which then enters the cytosol, where it is a required component for inflammasome formation. In the heart, calpain activation occurs at reperfusion. Calpain translocates to the cardiac membranes during ischemia [18,31], placing it near any bound procaspase-1 so that it would be in position to free procaspase-1 when Ca^++^ enters during reperfusion. Blocking calcium entry with a Na^+^/Ca^++^ exchange inhibitor or providing a calpain blocker equally protects hearts [18]. Indeed, the silencing of calpain expression was recently reported to protect the heart from I/R injury by suppressing the activation of the NLRP3/ASC/caspase-1 axis [32]. However, no mechanisms, such as blocking the release of bound procaspase-1, were discussed. 

The NLRP3 inflammasome may not even be necessary for caspase-1 activation. Zhang et al. [15] noted that when calpain released bound procaspase-1 in 293 cells that lacked inflammasome components, it still produced active caspase-1 through autoactivation. Calpain-dependent procaspase-1 release could cause the appearance of active caspase-1 through autoactivation in cardiomyocytes with the onset of reperfusion, independent of NLRP3, and could explain why the exact role of NLRP3 in reperfusion injury has been controversial. 

There are several limitations to our experimental protocols. Perhaps the most obvious is our use of heterozygous AC10^−/+^ hearts in some of our pharmacologic substudies. Because our breeding program was initiated to produce homozygous AC10^−/−^ knockout mice, the production of many otherwise unwanted heterozygous littermates was inevitable. Rather than automatically euthanizing these heterozygotes, we determined whether they could be used in our studies. Although the heterozygotes were missing one copy of the AC10 gene, the complement of cellular AC10 was normal, and there were no phenotypic features associated with the absence of the one gene. Furthermore, and most importantly, IS after I/R was similar in wild-type, heterozygous AC10^−/+^, and AC10^−/−^ knockout mice. Hence, rather than euthanizing these heterozygotes, we felt they would be adequate for our infarct studies. Of course, we cannot unequivocally exclude any subtle influence of a single missing AC10 gene on IS.

Second, we started our studies with male wild-type mice and continued with male AC10^−/+^ heterozygotes. However, male caspase-1/4-knockout mice were in short supply. This forced us to use female caspase-1/4-knockouts for our infarct studies. Others have found no difference in IS between isolated and untreated male and female WT murine hearts [33]. Furthermore, male and female caspase-1/4-knockout hearts responded with similar infarct sizes in all groups studied. Therefore, sex did not appear to be an independent determinant of IS, thus enabling us to combine male and female caspase-1/4-knockout mice in our groups.

Our studies convincingly demonstrate the efficacy of caspase-1/4 antagonists in minimizing IS in a murine model of I/R. It is critical to realize that this protection occurs in the early minutes of reperfusion, thus dictating that the protective intervention must be applied shortly before reperfusion or in the first minutes of reflow. It is encouraging that the beneficial effects of caspase-1/4 blockade can be added to those of conditioning interventions (e.g., IPC and P2Y_12_ inhibition). Finally, calpain inhibition at reperfusion blocks caspase activation and thus also represents an infarct-sparing intervention. These observations provide hope that a clinically suitable protocol can be developed that would minimize infarction at the time of percutaneous angioplasty in patients with ST-segment elevation myocardial infarction.

Reperfusion injury has been studied for 35 years, and a number of interventions have been reported to reduce infarct size in animal models. However, no strategy except early reperfusion has yet been identified to reduce infarct size and improve outcomes in the clinical arena. Clearly, a cardioprotective intervention is still an unmet clinical need. There are multiple possible reasons for this failure, many of which have been summarized by Lecour et al. [34]. Briefly, possible explanations for why anti-infarct interventions that were so highly protective in animals failed in clinical trials include the following: protection was blocked by comorbidities, the heart was already protected by one or more of the many administered drugs, the protection was blocked by one or more of the administered drugs, the long ischemic time in patients makes the benefit too small to measure, and there is a fundamental difference between human and animal hearts. One of our important observations is that multiple mechanisms contribute to the ultimate infarct size. Here, we show that ischemic preconditioning and caspase-1 inhibition are equally protective and, when combined, yield additive protection. These two interventions obviously are blocking two distinct sources of infarction. Other interventions have been reported to reduce infarct size, suggesting that there may be other preventable processes contributing to reperfusion injury. Recently, aspirin, which has no direct effect on infarct size, has been noted to block postconditioning’s protection in rat hearts [9]. Since all patients undergoing reperfusion therapy currently receive a loading dose of aspirin, and because most interventions that have been tested protect via the conditioning mechanism, this could be the simple explanation for the observed past failures. The solution would be to provide a postconditioning intervention without aspirin or use a caspase-1 inhibitor that protects by a different mechanism than postconditioning and is likely not aspirin sensitive or, better yet, do both.

## 4. Methods and Materials

### 4.1. Infarct Size Model

In accordance with published guidelines [35] and regulations of the local Institutional Animal Care and Use Committee, mice were anesthetized with 100 mg/kg sodium pentobarbital injected intraperitoneally. The trachea was exposed through a ventral cervical incision, intubated, and connected to a respirator that inflated the lungs with 100% oxygen under positive pressure. The heart was then exposed through a left thoracotomy and carefully excised, leaving several mm of aortic root. The aortic root was quickly cannulated (ischemic time < 4 min), and the hearts were mounted on a Langendorff perfusion apparatus to allow retroperfusion of the heart at a constant pressure of 78 mmHg, with modified Krebs-Henseleit bicarbonate buffer containing (in mM): 118.5 NaCl, 24.7 NaHCO_3_, 4.7 KCl, 2.4 MgSO_4_, 1.2 KH_2_PO_4_, 2.3 CaCl_2_, and 10.0 glucose. The buffer was saturated with 95% oxygen/5% carbon dioxide at 37 °C. The hearts were allowed to beat spontaneously without pacing, and the ventricles were empty. Coronary flow was monitored by measuring the volume of a timed collection of buffer dripping from the heart before coronary occlusion and at 30 min intervals after reperfusion. All hearts experienced a 20 min stabilization period before any ischemic intervention. Perfusion was stopped for 50 min to create global ischemia. During that period, the heart was immersed in buffer in a water-jacketed cup maintained at 37 °C. The heart was then reperfused for 2 h. 

At the end of reperfusion, the heart was removed and quickly frozen. The frozen heart was then sliced into ~1.2 mm thick slices from apex to base. These were incubated in 1% triphenyltetrazolium chloride heated to 37 °C for 20 min to stain the surviving tissue. Slices were arranged and then sandwiched between two glass plates held exactly 1 mm apart by shims. 

**Figure 4:**
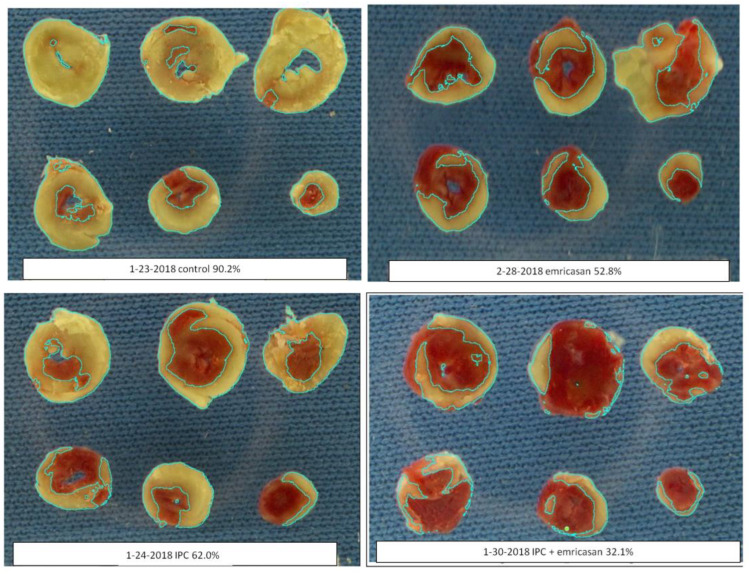
Representative examples of four typical hearts from protocol I (see below) analyzed by ImageJ. The six slices from the heart are stained with tetrazolium. The infarct (pale area) is outlined with a line as drawn by ImageJ’s color threshold function. The same threshold settings were used for all hearts. The white paper strip at the bottom of each panel is a 2 cm long scale bar.

The slices were photographed on a blue background with a high-resolution Pentax digital camera. A 2 cm white tab was included in the field of the photograph so that the size of an individual pixel could be calibrated. The images were analyzed with ImageJ 1.52a software using the color threshold command. We found that with our exposure, a “Hue” of 23–88, “Saturation” of 82–255, and “Brightness” of 89–255 using the default HSB configuration provided a sharp differentiation of the infarcted tissue (tan) from the surviving tissue (stained red). The pixel count of the infarcted tissue was measured. Changing “Hue” to 0–88 and adjusting “Brightness” as needed resulted in selection of all the tissue (i.e., the risk region), and the pixel count was measured again. The percentage of the heart that was infarcted was determined by dividing the number of pixels in the infarcted regions by the number of pixels in all the tissue and multiplying by 100. The size of an individual pixel was calculated from the 2 cm tab in the photo, allowing us to calculate the volume of the total tissue and the infarct since the thickness of each compressed slice was exactly 1 mm. Because of the uniform thickness of the slices, area measurements were proportional to volume, and thus the % infarction could be calculated from the area measurements alone. Since the computer operator used the same ImageJ settings for all heart slice images, there was no need to blind the operator to the experimental conditions. Measurements from all hearts that were subjected to staining contributed to the final database. Figure 4 shows examples of stained and processed hearts analyzed by ImageJ.

### 4.2. Wild-Type (WT) Mice

Wild-type mice were bred in-house from C57BL/6 mice obtained from Charles River Laboratories. 

### 4.3. AC10 Knockout (KO) Mice

We obtained sAC-C2KO AC10^−/+^ males and females of the same genotype as C57BL/6 founders that were used in a study by Chen et al. [36] from J. Chen and J. Buck. Male AC10 KO mice are sterile. Therefore, to generate homozygous AC10 KO mice, we had to breed heterozygous male and female mice and select homozygous offspring by genotyping. Briefly, the murine sAC gene was disrupted in embryonic stem cells (ES cells) by homologous recombination. A replacement vector was designed to delete exons 2–4 of the AC10 wild-type locus, leaving both the splice acceptor site of exon 2 and the splice donor site of exon 4 intact. The deleted sequence was replaced with a LacZ/MC1-Neo^r^ selection cassette to provide a reporter gene [36]. 

Breeding using heterozygous male mice resulted in a large number of unwanted heterozygous male mice offspring that were destined to be culled. Our guidelines ask that we try to minimize the amount of unnecessary killing of laboratory animals. Because no effect on infarct size was seen in hetero- or homozygous mice (see above), we used the excess heterozygous male mice for many of the pharmacological studies presented above.

### 4.4. Caspase-1/4-Deficient Mice

Founders were obtained from the Jackson Laboratory (B6N.129S2-*Casp1^tm1Flv^*/J). This strain, relevant to studies of inflammation and cell death, carries a knockout allele of the caspase-1 gene as well as an incidental caspase-4 deficiency that unknowingly was present in the 129S2/SvPas-derived ES cells that were used to develop the caspase-1/4-KO. Unfortunately, caspases-1 and -4 are both capable of initiating pyroptosis and often act in concert with each other. Since neither of our pharmacological tools is capable of distinguishing between the two caspases, we have referred to them as capase-1/4.

### 4.5. NLRC4 KO Mice

Homozygous NLRC4 KO founders [37] were graciously provided by Dr. Fayyaz Sutterwala and bred in-house. NLRC4-deficient founders had been backcrossed onto the C57BL/6 genetic background for at least six generations. 

### 4.6. Protocol I

**Figure 5:**
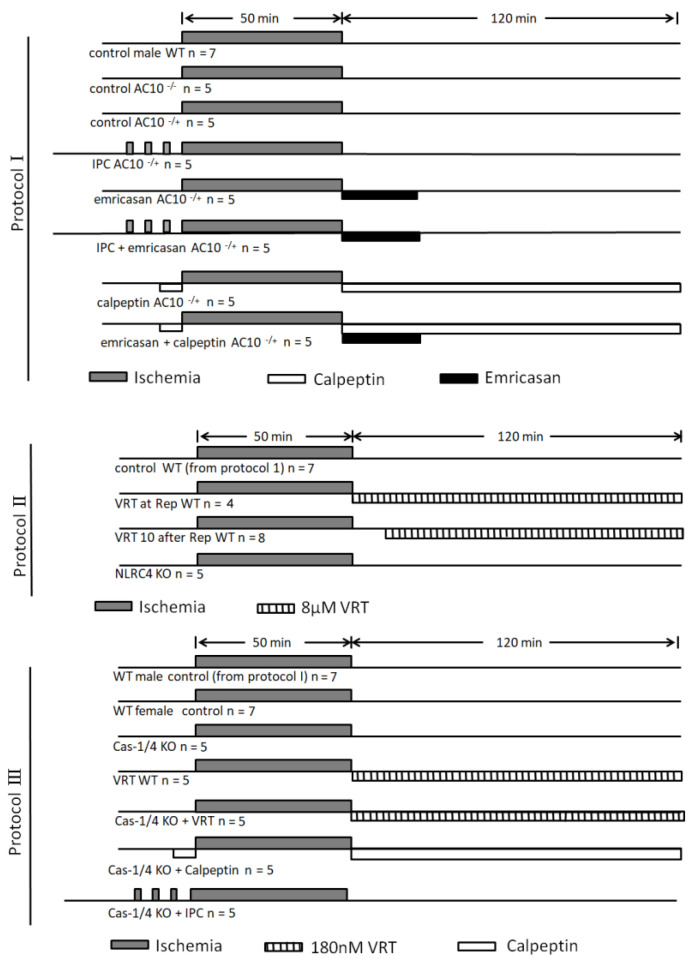
Diagram of the three protocols used in the study.

Figure 5 shows all protocols in graphic form. Isolated mouse hearts in protocol I were subjected to 50 min of global ischemia by stopping retrograde aortic flow to induce infarction, followed by 2 h of reperfusion. Hearts from untreated (control) WT C57BL/6 mice, AC10^−/−^ KO mice, and AC10^−/+^ heterozygous mice were studied, and there was no difference in infarct size among them (see Figure 1). Heterozygous AC10^−/+^ mice, which otherwise would have been needlessly culled, were used for cohorts 4 through 8. The heterozygous hearts had normal AC10 expression, with no detectable phenotype, and their response to ischemia was indistinguishable from that of WT hearts.

The fourth cohort in protocol I was ischemically preconditioned with three cycles of 5 min of no perfusion/5 min of reperfusion prior to the index ischemia. The fifth cohort was treated with the pan-caspase inhibitor emricasan (8 μM) for the first 20 min of reperfusion. In a monocyte cell assay, apoptosis-related caspases were inhibited with an EC_50_ of 0.27 µM [38]. An irreversible inhibitor has two determinants of its potency: the first is the affinity of the inhibitor for the enzyme (K_i_), and the second is the rate at which the bound enzyme is disabled (K_3_) [39]. Thus, its potency becomes a function of both its concentration and exposure time. The ratio of K_3_/K_i_ describes this potency. A higher ratio indicates greater potency. The ratio for caspase-1 is higher than that for caspase-3 and caspase-6 but lower than that for caspase-8. We used a concentration 30 times that used in the monocyte assay [38] to ensure not only that caspase-1 inhibition would occur but also that it would occur very soon after the beginning of the infusion. The sixth cohort was treated with both IPC and emricasan. The seventh cohort received the calpain inhibitor calpeptin at 1.38 µM starting 10 min prior to ischemia and continuing through reperfusion. In the eighth cohort, calpeptin and emricasan were combined.

### 4.7. Protocol II

Five new groups were studied in protocol II. Male WT mice provided hearts for four groups, and the fifth group consisted of NLRC4 knockouts. The first cohort of control WT mice was from protocol I and is added here for comparison. The second cohort of WT mice was reperfused with buffer containing the caspase-1 inhibitor VRT (8 µM). The third WT cohort was initially reperfused with drug-free buffer for 10 min and then switched to VRT-containing buffer for the remainder of the study. A final group of NLRC4 genetically deficient hearts was subjected to I/R.

### 4.8. Protocol III

For this protocol, we used our stock WT mice of both sexes. The control male WT hearts that were used in protocol 1 also served as our first cohort for protocol III. A group of female WT mouse hearts underwent the same 50 min ischemia/2 h reperfusion as the male hearts in cohort 1 and served as our second cohort. Infarcts in the female WT hearts were no different from those in the male WT hearts (see Figure 3). The absence of any difference in infarct size between sexes allowed us to use both male and female hearts for the caspase-1/4-deficient mice cohorts 3 and 5–7 since male caspase-1/4 KO mice were in very short supply. All cohorts underwent 50 min of global ischemia and 2 h of reperfusion. The female caspase-1/4-deficient hearts of the third cohort received no other treatment. A fourth cohort of mixed male and female WT hearts experienced the same I/R protocol except they were reperfused with buffer containing 180 nM VRT. Note that the concentration of VRT was lower than that used in protocol II. A recalculation based on the K_i_ reported by Boxer et al. [4] revealed that caspase-1 and caspase-4 could be blocked at a much lower concentration than we had been using. A fifth cohort of caspase-1/4-deficient hearts was reperfused with buffer containing VRT, as in the fourth cohort. A sixth cohort of caspase-1/4-deficient hearts was reperfused with calpeptin at the same concentration used in protocol I. A seventh cohort of caspase-1/4-deficient hearts underwent IPC, as described in protocol I, prior to ischemia.

### 4.9. Statistics

Infarct size was determined as the percentage of the entire left ventricle that was tetrazolium negative for each heart. Means and standard errors were determined for all 17 groups in the three protocols. Comparisons of infarct sizes among the 17 groups were made with a single one-way analysis of variance (ANOVA), which passed the Shapiro–Wilk normality test. Pairwise comparisons among our many groups were made using the Holm–Šidák method with Sigmaplot 12 software. While only one ANOVA test was performed for all 17 groups, we have displayed the results as three separate cohorts (Figure 1, Figure 2 and Figure 3) to aid in the comprehension of the results. To facilitate visual comparison of group differences, the same control group of male WT mice was included in each of the three cohorts. However, all *p*-values for the pairwise comparisons are those calculated on the basis of that single ANOVA with post hoc Holm–Šidák testing. All ANOVA and post hoc testing results are included in the supplement, as well as a figure presenting means and standard errors of all 17 groups.

## Data Availability

Infarct images available upon request. Other data contained within the article or supplementary material.

## Supplementary Materials

The supporting information can be downloaded at: https://www.mdpi.com/article/10.3390/ijms24043801/s1.
