# Supplementary material for: Lethal Caspase-1/4-Dependent Injury Occurs in the First Minutes of Coronary Reperfusion and Requires Calpain Activity
Source: Int J Mol Sci. 2023 Feb 14;24(4):3801. doi: 10.3390/ijms24043801 (PMC9960231; doi:10.3390/ijms24043801)
Supplement: Supplementary file 1 [file ijms-24-03801-s001.zip › ijms-2132863-supplementary.pdf]

One Way Analysis of Variance Wednesday, January 25, 2023, 10:31:17 AM

Data source: Data 1 in Notebook1

Dependent Variable: Col 1

Normality Test (Shapiro-Wilk) Passed (P = 0.096)

Equal Variance Test: Passed (P = 0.473)

Group Name	N	Missing	Mean	Std Dev	SEM
ACHET	5	0	88.340	5.209	2.330
IPC	5	0	59.300	10.216	4.569
IPCEM	5	0	26.980	5.779	2.584
EM	5	0	54.760	12.158	5.437
ACKO	5	0	87.796	2.332	1.043
MWT	7	0	87.217	4.539	1.716
VRTREP	4	0	45.300	7.052	3.526
VRT10m	8	0	72.638	12.589	4.451
CAL	5	0	57.420	11.230	5.022
CALEM	5	0	50.860	7.995	3.575
NLRC4KO	5	0	89.360	2.510	1.122
FWT	7	0	83.324	6.714	2.538
CAKO	5	0	42.960	9.852	4.406
WTVRT	5	0	43.700	10.832	4.844
CAKOVRT	5	0	44.140	9.743	4.357
CAKOCAL	5	0	45.240	10.849	4.852
CAKOIPC	5	0	18.240	6.981	3.122

Source of Variation	DF	SS	MS	F	P
Between Groups	16	43194.674	2699.667	35.587	<0.001
Residual	74	5613.681	75.861		
Total	90	48808.355			

The differences in the mean values among the treatment groups are greater than would be expected by chance; there is a statistically significant difference (P = <0.001).

Power of performed test with alpha = 0.050: 1.000

All Pairwise Multiple Comparison Procedures (Holm-Sidak method):
Overall significance level = 0.05

Comparisons for factor: **Col 2**

Comparison	Diff of Means	t	P	P<0.050
MWT vs. CAKOIPC	68.977	13.525	<0.001	Yes
NLRC4KO vs. CAKOIPC	71.120	12.911	<0.001	Yes
FWT vs. CAKOIPC	65.084	12.762	<0.001	Yes
ACHET vs. CAKOIPC	70.100	12.726	<0.001	Yes
ACKO vs. CAKOIPC	69.556	12.627	<0.001	Yes
MWT vs. IPCEM	60.237	11.811	<0.001	Yes
NLRC4KO vs. IPCEM	62.380	11.324	<0.001	Yes
ACHET vs. IPCEM	61.360	11.139	<0.001	Yes
FWT vs. IPCEM	56.344	11.048	<0.001	Yes
ACKO vs. IPCEM	60.816	11.040	<0.001	Yes
VRT10m vs. CAKOIPC	54.398	10.956	<0.001	Yes
VRT10m vs. IPCEM	45.658	9.195	<0.001	Yes
MWT vs. CAKO	44.257	8.678	<0.001	Yes
MWT vs. WTVRT	43.517	8.533	<0.001	Yes
MWT vs. CAKOVRT	43.077	8.447	<0.001	Yes
NLRC4KO vs. CAKO	46.400	8.423	<0.001	Yes
NLRC4KO vs. WTVRT	45.660	8.289	<0.001	Yes
ACHET vs. CAKO	45.380	8.238	<0.001	Yes
MWT vs. CAKOCAL	41.977	8.231	<0.001	Yes

NLRC4KO vs. CAKOVRT	45.220	8.209	<0.001	Yes
ACKO vs. CAKO	44.836	8.139	<0.001	Yes
ACHET vs. WTVRT	44.640	8.104	<0.001	Yes
ACHET vs. CAKOVRT	44.200	8.024	<0.001	Yes
NLRC4KO vs. CAKOCAL	44.120	8.009	<0.001	Yes
ACKO vs. WTVRT	44.096	8.005	<0.001	Yes
ACKO vs. CAKOVRT	43.656	7.925	<0.001	Yes
FWT vs. CAKO	40.364	7.915	<0.001	Yes
ACHET vs. CAKOCAL	43.100	7.824	<0.001	Yes
FWT vs. WTVRT	39.624	7.770	<0.001	Yes
ACKO vs. CAKOCAL	42.556	7.725	<0.001	Yes
FWT vs. CAKOVRT	39.184	7.683	<0.001	Yes
MWT vs. VRTREP	41.917	7.678	<0.001	Yes
NLRC4KO vs. VRTREP	44.060	7.541	<0.001	Yes
FWT vs. CAKOCAL	38.084	7.468	<0.001	Yes
IPC vs. CAKOIPC	41.060	7.454	<0.001	Yes
ACHET vs. VRTREP	43.040	7.366	<0.001	Yes
ACKO vs. VRTREP	42.496	7.273	<0.001	Yes
MWT vs. CALEM	36.357	7.129	<0.001	Yes
CAL vs. CAKOIPC	39.180	7.113	<0.001	Yes
NLRC4KO vs. CALEM	38.500	6.989	<0.001	Yes
FWT vs. VRTREP	38.024	6.965	<0.001	Yes
ACHET vs. CALEM	37.480	6.804	<0.001	Yes
ACKO vs. CALEM	36.936	6.705	<0.001	Yes
EM vs. CAKOIPC	36.520	6.630	<0.001	Yes
FWT vs. CALEM	32.464	6.366	<0.001	Yes
MWT vs. EM	32.457	6.364	<0.001	Yes
NLRC4KO vs. EM	34.600	6.281	<0.001	Yes
ACHET vs. EM	33.580	6.096	<0.001	Yes
ACKO vs. EM	33.036	5.997	<0.001	Yes
VRT10m vs. CAKO	29.678	5.977	<0.001	Yes
CALEM vs. CAKOIPC	32.620	5.922	<0.001	Yes
IPC vs. IPCEM	32.320	5.867	<0.001	Yes
MWT vs. CAL	29.797	5.843	<0.001	Yes
VRT10m vs. WTVRT	28.938	5.828	<0.001	Yes
NLRC4KO vs. CAL	31.940	5.798	<0.001	Yes
VRT10m vs. CAKOVRT	28.498	5.739	<0.001	Yes
ACHET vs. CAL	30.920	5.613	<0.001	Yes
FWT vs. EM	28.564	5.601	<0.001	Yes
CAL vs. IPCEM	30.440	5.526	<0.001	Yes
VRT10m vs. CAKOCAL	27.398	5.518	<0.001	Yes
ACKO vs. CAL	30.376	5.514	<0.001	Yes
MWT vs. IPC	27.917	5.474	<0.001	Yes
NLRC4KO vs. IPC	30.060	5.457	<0.001	Yes
ACHET vs. IPC	29.040	5.272	<0.001	Yes
ACKO vs. IPC	28.496	5.173	<0.001	Yes
VRT10m vs. VRTREP	27.338	5.126	<0.001	Yes
FWT vs. CAL	25.904	5.079	<0.001	Yes
EM vs. IPCEM	27.780	5.043	<0.001	Yes
CAKOCAL vs. CAKOIPC	27.000	4.901	<0.001	Yes
FWT vs. IPC	24.024	4.711	<0.001	Yes
CAKOVRT vs. CAKOIPC	25.900	4.702	<0.001	Yes
VRTREP vs. CAKOIPC	27.060	4.631	<0.001	Yes
WTVRT vs. CAKOIPC	25.460	4.622	0.001	Yes
CAKO vs. CAKOIPC	24.720	4.488	0.002	Yes
VRT10m vs. CALEM	21.778	4.386	0.002	Yes
CALEM vs. IPCEM	23.880	4.335	0.003	Yes
VRT10m vs. EM	17.878	3.601	0.034	Yes
NLRC4KO vs. VRT10m	16.722	3.368	0.069	No
CAKOCAL vs. IPCEM	18.260	3.315	0.079	No
MWT vs. VRT10m	14.579	3.234	0.099	No
ACHET vs. VRT10m	15.702	3.162	0.119	No
VRTREP vs. IPCEM	18.320	3.136	0.127	No
CAKOVRT vs. IPCEM	17.160	3.115	0.132	No

VRT10m vs. CAL	15.218	3.065	0.149	No
ACKO vs. VRT10m	15.158	3.053	0.151	No
WTVRT vs. IPCEM	16.720	3.035	0.156	No
IPC vs. CAKO	16.340	2.966	0.184	No
CAKO vs. IPCEM	15.980	2.901	0.214	No
IPC vs. WTVRT	15.600	2.832	0.249	No
IPC vs. CAKOVRT	15.160	2.752	0.296	No
VRT10m vs. IPC	13.338	2.686	0.338	No
CAL vs. CAKO	14.460	2.625	0.379	No
IPC vs. CAKOCAL	14.060	2.552	0.432	No
CAL vs. WTVRT	13.720	2.491	0.478	No
CAL vs. CAKOVRT	13.280	2.411	0.542	No
IPC vs. VRTREP	14.000	2.396	0.546	No
FWT vs. VRT10m	10.686	2.371	0.561	No
CAL vs. CAKOCAL	12.180	2.211	0.697	No
EM vs. CAKO	11.800	2.142	0.747	No
CAL vs. VRTREP	12.120	2.074	0.792	No
EM vs. WTVRT	11.060	2.008	0.832	No
EM vs. CAKOVRT	10.620	1.928	0.875	No
EM vs. CAKOCAL	9.520	1.728	0.957	No
EM vs. VRTREP	9.460	1.619	0.978	No
IPCEM vs. CAKOIPC	8.740	1.587	0.981	No
IPC vs. CALEM	8.440	1.532	0.987	No
CALEM vs. CAKO	7.900	1.434	0.994	No
CALEM vs. WTVRT	7.160	1.300	0.998	No
CALEM vs. CAKOVRT	6.720	1.220	0.999	No
CAL vs. CALEM	6.560	1.191	0.999	No
NLRC4KO vs. FWT	6.036	1.183	0.999	No
CALEM vs. CAKOCAL	5.620	1.020	1.000	No
ACHET vs. FWT	5.016	0.983	1.000	No
CALEM vs. VRTREP	5.560	0.952	1.000	No
ACKO vs. FWT	4.472	0.877	1.000	No
MWT vs. FWT	3.893	0.836	1.000	No
IPC vs. EM	4.540	0.824	1.000	No
EM vs. CALEM	3.900	0.708	1.000	No
CAL vs. EM	2.660	0.483	1.000	No
NLRC4KO vs. MWT	2.143	0.420	1.000	No
CAKOCAL vs. CAKO	2.280	0.414	1.000	No
VRTREP vs. CAKO	2.340	0.400	1.000	No
IPC vs. CAL	1.880	0.341	1.000	No
NLRC4KO vs. ACKO	1.564	0.284	1.000	No
CAKOCAL vs. WTVRT	1.540	0.280	1.000	No
VRTREP vs. WTVRT	1.600	0.274	1.000	No
ACHET vs. MWT	1.123	0.220	1.000	No
CAKOVRT vs. CAKO	1.180	0.214	1.000	No
CAKOCAL vs. CAKOVRT	1.100	0.200	1.000	No
VRTREP vs. CAKOVRT	1.160	0.199	1.000	No
NLRC4KO vs. ACHET	1.020	0.185	1.000	No
WTVRT vs. CAKO	0.740	0.134	1.000	No
ACKO vs. MWT	0.579	0.114	1.000	No
ACHET vs. ACKO	0.544	0.0987	1.000	No
CAKOVRT vs. WTVRT	0.440	0.0799	0.996	No
VRTREP vs. CAKOCAL	0.0600	0.0103	0.992	No

Key: ACHET= AC10 heterozygote (het), IPC = Ischemic preconditioning of AC10 het, IPCEM = Ischemic preconditioning + emricasan in AC10 het, EM = Emricasan in AC10 het, ACKO = AC10 knockout, MWT = male wild type, VRTREP = 8 µM VRT started at reperfusion in male wild type, VRT10m = 8 µM VRT started 10 min after the onset of reperfusion in male wild type, CAL = Calpeptin in AC10 het, CALEM = Calpeptin + emricasan in AC10 het, NLRC4KO = Male NLRC4 knockout, FWT = Female wild type, CAKO = Female caspase-1/4 knockout, WTVRT = 180 nM VRT at reperfusion in mixed gender wild type mice, CAKOVRT = 180 nM VRT at reperfusion in mixed

gender caspase-1/4 knockout, CAKOCAL = Calpeptin in mixed gender caspase-1/4 knockout, CAKOIPC = Ischemic preconditioning in mixed gender caspase-1/4 knockout

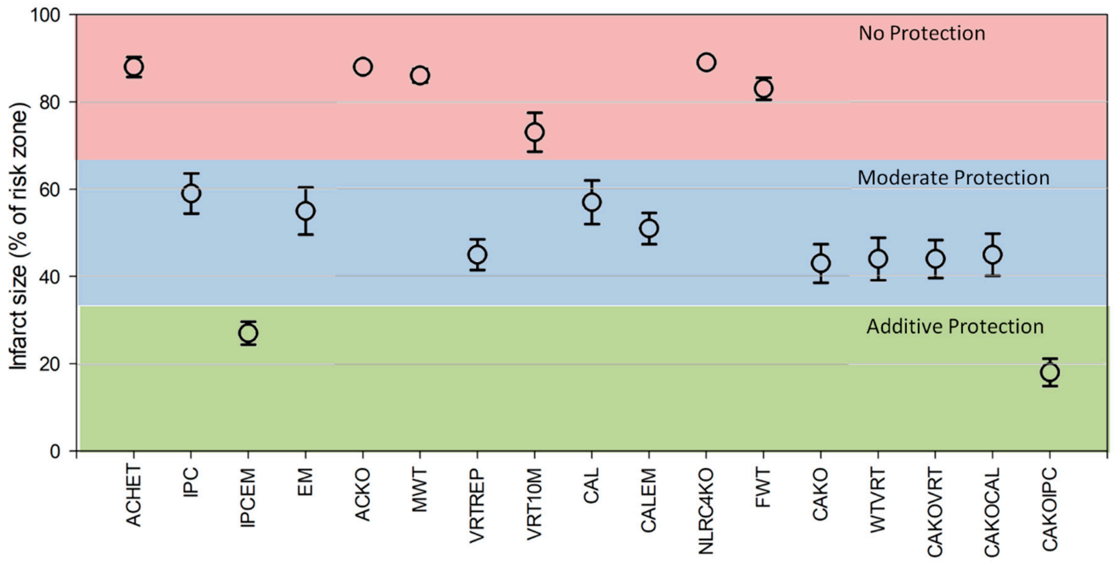

In this figure the three colored zones reflect degrees of protection experienced by the experimental groups. In the top pink zone there is no protection, and infarct sizes of all groups in this zone are not statistically different. The groups in the middle blue zone demonstrate moderate protection. Infarct sizes of all groups in this zone are not different from each other, but are statistically different from those in the two other zones. The bottom green zone encompasses groups that have displayed additive protection when two interventions have been combined. Again, infarct sizes in these groups are not different, but they are significantly different from groups in either of the two other zones. All group names are defined as indicated above.
